# Estimation and Control of Cooperative Aerial Manipulators for a Payload with an Arbitrary Center-of-Mass

**DOI:** 10.3390/s21196452

**Published:** 2021-09-27

**Authors:** Hyeonbeom Lee, Uikyum Kim

**Affiliations:** 1 School of Electronic and Electrical Engineering, Kyungpook National University, Daegu 41566, Korea; hbeomlee@knu.ac.kr; 2 Department of Mechanical Engineering, Ajou University, Suwon 16499, Korea

**Keywords:** parameter estimation, arbitrary center-of-mass, cooperative aerial manipulation, dynamic uncertainty

## Abstract

This paper presents an integrated framework that integrates the kinematic and dynamic parameter estimation of an irregular object with non-uniform mass distribution for cooperative aerial manipulators. Unlike existing approaches, including impedance-based control which requires expensive force/torque sensors or the first-order-momentum-based estimator which is weak to noise, this paper suggests a method without such sensor and strong to noise by exploiting the decentralized dynamics and sliding-mode-momentum observer. First, the kinematic estimator estimates the relative distances of multiple aerial manipulators by using translational and angular velocities between aerial robots. By exploiting the distance estimation, the desired trajectories for each aerial manipulator are set. Second, the dynamic parameter estimation is performed for the mass of the common object and the vector between the end-effector frame and the center of mass of the object. Finally, the proposed framework is validated with simulations using aerial manipulators combined with two degrees-of-freedom robotic arms using a noisy measurement. Throughout the simulation, we can decrease the mass estimation error by 60% compared to the existing first-order momentum-based method. In addition, a comparison study shows that the proposed method satisfactorily estimates an arbitrary center-of-mass of an unknown payload in noisy environments.

## 1. Introduction

Multirotor UAVs (unmanned aerial vehicles), which are highly maneuverable and can be made small, are gaining popularity as a major air transportation platform [1]. Among them, cooperative UAVs are widely exploited to handle a heavy or large payload [2] beyond the limits of a robot’s transportation capabilities. Recently, researchers have developed cooperative mobile manipulators [2,3,4,5,6,7,8,9] by exploiting grasping capability. However, due to several issues including the complexity associated with multiple aerial robots, they have focused on solving a control and coordination problem. To make the problem simple, they have assumed that a relative distance from the payload frame can be easily calculated because the regular payload has a uniform mass distribution [2,4,5,6,7,8,9]. For these reasons, cooperative aerial manipulations to handle a payload with the non-uniform mass distribution (i.e., the geometry of the payload and center of mass (COM) of the payload are different) are quite complicated.

### 1.1. Contribution

This paper is interested in resolving the aforementioned problem of handling a payload with an arbitrary center-of-mass for cooperative aerial manipulators. The contributions of this paper can be summarized as follows. First, we propose a kinematic estimation algorithm for unknown kinematics of the payload and the trajectory generation algorithm using the estimated kinematic parameter. The existing method in [10] estimates the relative kinematic parameter between each end-effector. However, the proposed algorithm can estimate not only the kinematic parameters, but also the the coordinates of COM of the payload, which is crucial for handling a payload with an arbitrary COM. By exploiting the distance estimation, the desired trajectories for each aerial manipulator are set. Second, we propose a dynamic parameter estimation for the mass and COM of a payload by using the external wrench estimation algorithm. While existing approaches require a force/torque sensor [11] or a vision sensor [12], the proposed algorithm does not require such sensors. Unlike the existing estimation algorithms for estimating unknown kinematic and dynamic parameters [9,13], the proposed algorithm does not require an assumption of uniform mass distribution or contact-force control. In addition, the proposed algorithm shows better estimation performance of dynamic parameters even with a noisy measurement than the first-order momentum-based approach [14,15,16] or our previous research [4]. Finally, the numerical simulation results show that the feasibility of the proposed algorithm is capable of allowing cooperative aerial manipulators to jointly carry a payload with an arbitrary center-of-mass.

### 1.2. Related Works

Early works on the cooperative aerial manipulation assumed that the dynamic and kinematic information of the payload is known [2]. They designed a fully centralized controller and planning algorithm with a known payload model. Unlike these centralized approaches, in [11], the decentralized impedance controller was applied for cooperative aerial manipulators. The mass and COM of the payload were estimated by the external wrench, which is measured by the force-torque sensor. In [17], the authors designed an optimization-based force decomposition algorithm and decentralized force controller with a payload stiffness model. However, these methods require expensive force-torque sensors, which are difficult to be equipped in aerial robots because of a payload limitation. Contact force estimation algorithm for dual-arm aerial manipulator was developed using the Cartesian deflection of the end-effector measured by a stereo vision sensor in [12]. Although the method shows precise estimation performance of the payload mass, the stereo camera always watches the end-effector for the deflection measurement.

Another approach is to design a controller that is robust/adaptive to parameter changes in the kinematic or dynamic payload model [4,6,7,8,18]. A robust optimal control is designed for dual arm manipulator in [18]. In [4,8], decentralized adaptive controllers were designed based on a consensus algorithm to ensure that the estimated mass from every agent gets an equal share of the mass of the payload. In [6], the robust controller with a disturbance observer is used for each aerial manipulator to carry a common payload. In [7], the effects of parametric uncertainties for each aerial manipulator were removed by designing a robust controller without using explicit communication between agents. While these methods [4,6,7,8] can easily handle the mass changes of the payload, the kinematic parameters should be known.

Kinematic parameter estimation for cooperative manipulations is used for ground manipulators [10] or human–robot interaction [19]. As the exact position of human or other manipulator is difficult to be identified in both applications, the kinematic parameter estimation is essential to complete the cooperative tasks. In [10], the relative kinematics between the robotic end-effectors can be identified based on the motion signals of the end-effector. In [19], the unknown kinematic and dynamic model of a payload are estimated for the human–robot manipulation tasks. For the aerial robots, this relative position may easily be estimated when they have an accurate global positioning sensor (GPS). However, the GPS sensor cannot be applied to GPS-denied environments. To resolve this issue, in [20], the relative position and orientation for rigidly-attached quadrotors were estimated without using an external force-torque sensor. However, this method only estimates kinematic parameters including the relative length and orientation of each quadrotor. Therefore, it is hard to apply for cooperative aerial manipulators which carry a payload with an arbitrary COM.

There exists some research for handling both the dynamic and kinematic uncertainties [9,13]. In [13], a distributed algorithm for estimating the kinematic and dynamic parameters of a common payload was proposed for cooperative ground mobile manipulators. However, this method assumes that each ground mobile manipulator can control the contact force, which is difficult to use for the aerial robots. In addition, they do not perform estimation and control simultaneously. In [9], they developed a two-stage approach: at the first stage, each robot estimates the payload kinematic and dynamic parameters using external wrenches, while, in the second stage, the estimated parameters are used for a distributed cooperative controller. Although the proposed algorithm can handle a payload with a arbitrary COM, this method [9] is only applicable to a payload with a uniform mass distribution.

## 2. Dynamics

**Figure 1:**
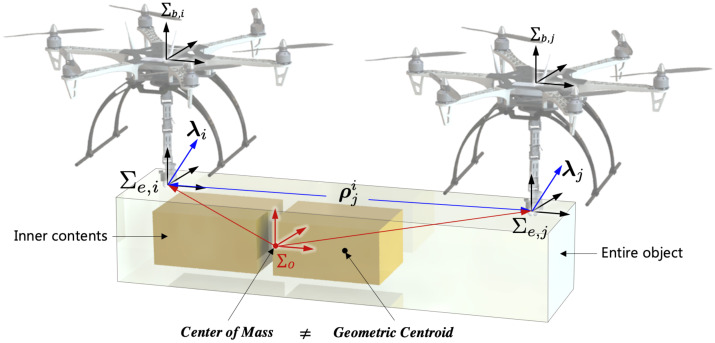
Cooperative flight with a payload with a variable CoM.

It is necessary to analyze the dynamics of aerial manipulators when they transport a payload with an arbitrary COM as shown in Figure 1. In this figure, the distance $\rho_{j}^{i}$ is the estimated relative distance to *j*-th end-effector by *i*-th aerial manipulator and $\tau_{i}^{b}$ is the external wrench. In general, we will use bold letters (e.g., $\tau_{i}^{b}$) to indicate vector quantities and the upper manuscript (e.g., e,b etc.) to indicate the frame.

The dynamics for cooperative aerial manipulators is addressed. Figure 1 shows that two cooperative aerial manipulators transport a payload with non-uniform mass distribution. The coordination $\Sigma_{e,i},\Sigma_{b,i}$, and $\Sigma_{o}$ are the end-effector frame, the body frame of each aerial manipulator, and the common payload frame, respectively. For better understanding, the definition of the used terms in this paper is summarized in Table 1.

### 2.1. Aerial Manipulator and Payload Dynamics

Using the position of the COM of the multirotor in $\Sigma_{I}$, we define the states of the *i*-th aerial manipulator $q_{i}\in R^{6+n}$ (i=1,...,N) in terms of the position of the multirotor UAV, Euler angle of the multirotor, and joint angles of a robotic arm. Here, n=2 is the number of degrees of freedom (DOFs) of a robotic arm and *N* represents the total number of aerial manipulators.

While carrying a common payload, the dynamics of the *i*-th the aerial manipulator can be obtained as

(1) $$\begin{array}{c} M_{i}\left(q_{i}\right){\ddot{q}}_{i}+Q_{i}\left(q_{i},{\dot{q}}_{i}\right){\dot{q}}_{i}+W_{i}\left(q_{i}\right)=\tau_{i}+\tau_{i}^{b}, \end{array}$$

where $M_{i}\in R^{8\times 8}$ is the inertia matrix, $Q_{i}\in R^{8\times 8}$ is the Coriolis matrix, and $W_{i}\in R^{8}$ is the gravity term. $\tau_{i}$ is the control input in the inertial frame $\Sigma_{I}$ and $\tau_{i}^{b}$ is the external wrench. In this study, we can set

(2) $$\begin{array}{c} \tau_{i}^{b}=-J_{i}^{T}\left(q_{i}\right)\lambda_{i} \end{array}$$

where $\lambda_{i}\in R^{6}$ is the wrench applied to the end-effector of the *i*-th aerial manipulator in $\Sigma_{e,i}$. $J_{i}\left(q_{i}\right)\in R^{6\times 8}$ means the Jacobian matrix from $\Sigma_{b,i}$ to $\Sigma_{e,i}$.

Then, the dynamics of a common payload can be defined as

(3) $$\begin{array}{c} H_{o}{\ddot{q}}_{o}+\mu_{o}{\dot{q}}_{o}+G_{o}=\sum_{i=1}^{N}E_{i}\lambda_{i}, \end{array}$$

where ${\dot{q}}_{o}\in R^{6}$ is the state of a payload. The state consists of a six-dimensional vector including the translational velocity and rotational velocity of the payload in $\Sigma_{I}$. We use $H_{o}$ = diag$\left(m_{o}I_{3},J_{o}\right)\in R^{6\times 6}$, where $m_{o}$ is the mass of the payload, $J_{o}\in R^{3\times 3}$ is the inertia, and $I_{3}$ is 3×3 identity matrix. The parameter $\mu_{o}\in R^{6\times 6}$ is a matrix containing centripetal terms and the Coriolis. $G_{o}\in R^{6}$ is a gravity matrix. $E_{i}\in R^{6\times 6}$ is a grasp matrix and is given by

(4) $$\begin{array}{c} E_{i}=\left[\begin{array}{cc} I_{3} & 0_{3}\\ S(r_{i}) & I_{3} \end{array}\right], \end{array}$$

where $0_{3}$ is a 3×3 zero matrix and $S(r_{i})$ is the skew-symmetric matrix expressing the cross-product from the position of $\Sigma_{o}$ for $\Sigma_{e,i}$.

### 2.2. Combined Dynamics

Using the assumption of a rigid grasp, the wrench $\lambda_{i}$ can be computed using the force distribution solution [21] as

(5) $$\begin{array}{c} \lambda_{i}=c_{i}E_{i}^{†}\left(H_{o}{\ddot{q}}_{o}+\mu_{o}{\dot{q}}_{o}+G_{o}\right), \end{array}$$

where $c_{i}$ is the weight satisfying $\sum_{i=1}^{N}$ and $E_{i}^{†}$ can be obtained using the Moore–Penrose pseudo-inverse solution as

(6) $$\begin{array}{c} c_{i}E_{i}^{†}=\left[\begin{array}{cc} c_{i}I_{3} & -c_{i}S\left(r_{i}\right)\Pi^{-1}\\ 0_{3} & c_{i}\Pi^{-1} \end{array}\right], \end{array}$$

with $\Pi =I_{3}+\sum_{i=1}^{N}c_{i}S\left(r_{i}\right)S^{T}\left(r_{i}\right)$. To compute Π, the position of all end-effector with respect to $\Sigma_{o}$ is required. In our previous work [22], we set $c_{i}=1/N$ and $r_{i}$ is computed based on the assumption of uniform mass distribution. However, in this paper, we estimate $r_{i}$ using the relative distance ρ and the estimated force applied to the end-effector. Using the estimated $r_{i}$, the weight value $c_{i}$ can be calculated.

As the manipulator grasps the paylod rigidly, all positions and orientations (i.e., $q_{o}$) of the payload and the end-effectors can be expressed using the state variable of the aerial manipulator itself (i.e., $q_{i}$). We can obtain the following equation as

(7) $$\begin{array}{cc} {\dot{q}}_{o} & =E_{i}^{-T}J_{i}{\dot{q}}_{i}. \end{array}$$

By substituting (5) and (7) into (1), we can obtain the decentralized motion equation for the *i*-th aerial manipulator as

(8) $$\begin{array}{c} D_{i}\left(q_{i}\right){\ddot{q}}_{i}+C_{i}\left(q_{i},{\dot{q}}_{i}\right){\dot{q}}_{i}+G_{i}\left(q_{i}\right)=\tau_{i}. \end{array}$$

Here, the matrices are calculated as

(9) $$\begin{array}{cc} D_{i} & =M_{i}\left(q_{i}\right)+c_{i}M_{o}\left(q_{i}\right),G_{i}=W_{i}+c_{i}W_{o}\left(q_{i}\right)\\ C_{i} & =Q_{i}\left(q_{i},{\dot{q}}_{i}\right)+c_{i}Q_{o}\left(q_{i},{\dot{q}}_{i}\right)+c_{i}J_{i}^{T}\left(E_{i}^{†}H_{o}E_{i}^{-T}\right){\dot{J}}_{i} \end{array}$$

where the following representations hold

(10) $$\begin{array}{cc} M_{o}\left(q_{i}\right) & =J_{i}^{T}\left(E_{i}^{†}H_{o}E_{i}^{-T}\right)J_{i}\in R^{8\times 8},\\ Q_{o}\left(q_{i},{\dot{q}}_{i}\right) & =J_{i}^{T}\left(E_{i}^{†}\mu_{o}E_{i}^{-T}\right)J_{i}\in R^{8\times 8},\\ W_{o}\left(q_{i}\right) & =J_{i}^{T}E_{i}^{†}G_{o}\in R^{8}. \end{array}$$

The detailed process for (10) are described in the previous work of this research [22]. Thanks to (8), we can control the aerial manipulator separately.

## 3. Kinematic Parameter Estimation and Path Planning

**Figure 2:**
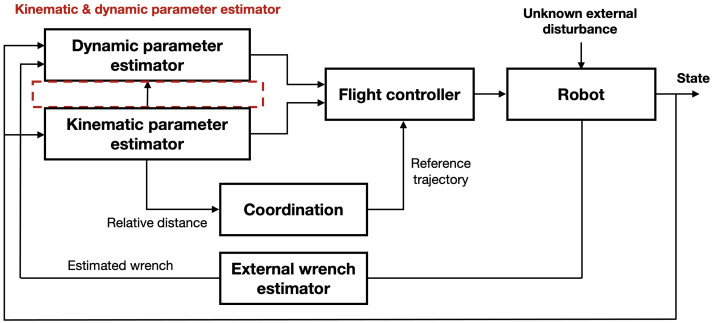
The proposed structure for handling a payload with a non-uniform mass distribution.

In this section, we address the kinematic parameter estimation and coordination algorithm for cooperative aerial manipulations. The overall structure is shown in Figure 2. The distance $\rho_{j}^{i}$, heading angle $\psi_{j}^{i}$ between *i*-th and *j*-th robots, and mass $m_{o}$ are estimated by an online parameter estimator by receiving the state $q_{i}$ and the estimated external wrench $\tau_{i}^{b}$. The estimated kinematic parameters are exploited to calculate the geometry centroid of the payload. With the given desired trajectory of the common payload, the desired trajectory for each aerial manipulator can be computed using the geometry centroid. After the estimation of the dynamic parameters $r_{i}$ and $m_{o}$ in (5), the controller generates the control input.

Estimation of the relative distance $\rho_{j}^{i}$ can be done with a vision sensor [23] for the indoor environment or RTK-GPS sensor [24] for the outdoor environment. However, the method [23] assumed that the vision sensor always watches the plane to compute the homography matrix. In the outdoor environment, the position can be measured by RTK-GPS with a few centimeters accuracies [24], but this system cannot be applied in indoor environments or near buildings. To overcome this issue, this paper proposes the kinematic parameter estimation algorithm only using a state variable itself.

### 3.1. Kinematic Parameter Estimation

To achieve this goal, we first make the following assumptions on the kinematic model of cooperative manipulators.

(1) The position/orientation of the end effector grasping on to the payload in the global frame are not known.
(2) At each grasping point, the roll and pitch angle of the end effector is the same for the cooperative aerial manipulators.
(3) Configurations of the payload are previously given, so aerial manipulators know the grasping point of the payload. However, the exact relative distances and heading angles between each robot are unknown.

For the ground manipulator, it is necessary to estimate the six DOF kinematic model (i.e., three DOF for the distance and three DOF for the relative attitude). However, for an aerial manipulator, roll (i.e., $\phi_{i}$) and pitch (i.e., $\theta_{i}$) angles can be computed without estimation because those angles are close to 0 degrees in the hovering state, or easily obtained on the IMU sensor. Despite the inertial measurement unit (IMU) sensor, it is impossible to accurately estimate the heading angle in the global frame even with the magnetometer sensor. Because of these issues, unlike approaches for ground manipulators [10,19], we only estimate the relative heading angle. Therefore, our approach is divided into two steps: (1) heading angle $\psi_{j}^{i}$, and (2) the relative distance $\rho_{j}^{i}$.

The relative heading angle is estimated from the measurement of the IMU sensor around the *z*-axis. From the assumption of rigid grasping, the module pair $\left[\phi_{i},\theta_{i}\right]$ has to be the same for both *i* and *j*-th aerial manipulator in the same time step *k* as

(11) $$\begin{array}{c} \frac{{}^{k}\theta_{i}}{{}^{k}\phi_{i}}=\frac{{}^{k}\theta_{j}}{{}^{k}\phi_{j}}\tan\left(\psi_{i}^{i}\right). \end{array}$$

The above equation should be satisfied for the whole process, so we can augment the Equation (11) as

(12) $$\begin{array}{c} {\hat{\psi }}_{j}^{i}=\frac{\sum_{k=1}^{K}\left(\tan^{-1}\left(\frac{{}^{k}\theta_{i}}{{}^{k}\phi_{i}}\right)-\tan^{-1}\left(\frac{{}^{k}\theta_{j}}{{}^{k}\phi_{j}}\right)\right)}{K}, \end{array}$$

where *K* is the total number of time steps. It is impossible to estimate the relative heading angle when the roll and pitch angles are exactly 0 degrees, but in the case of drones, this is not the case, so the algorithm can run.

For the relative distance estimation, we analyze the kinematics of the payload. Let $v_{i}^{e}$ and $\omega_{i}^{e}$ be the linear and angular velocities of the end-effector of the *i*-th aerial manipulator in $\Sigma_{e,i}$. These terms can be computed as

(13) $$\left[\begin{array}{c} v_{i}^{e}\\ \omega_{i}^{e} \end{array}\right]=\left[\begin{array}{cc} R_{i}^{T} & 0\\ 0 & T_{i} \end{array}\right]J_{i}{\dot{q}}_{i}$$

where $R_{i}$ is the rotation matrix, which represents the orientation of $\Sigma_{b,i}$ for the inertial frame. $T_{i}$ is a mapping matrix between the angular rate of an aerial robot in $\Sigma_{I}$ and the body angular rate $\omega_{i}^{e}$ in $\Sigma_{e,i}$.

The end-effector velocities at the *i*-th and *j*-th aerial manipulator should satisfy the following constraints:

(14) $$\begin{array}{cc} \omega_{i}^{e} & =R\left({\hat{\psi }}_{j}^{i}\right)\omega_{j}^{e}\\ v_{i}^{e} & =R\left({\hat{\psi }}_{j}^{i}\right)v_{j}^{e}-\rho_{j}^{i}\times \omega_{i}^{e} \end{array}$$

where $R({\hat{\psi }}_{j}^{i})$ is the rotation matrix using ${\hat{\psi }}_{j}^{i}$, which is the orientation of $\Sigma_{e,j}$ for $\Sigma_{e,i}$.

The proposed method for parameter estimation $\rho_{ij}$ is updated based on the following error terms

(15) $$\begin{array}{cc} e_{v_{i}} & =v_{i}^{e}-R\left({\hat{\psi }}_{j}^{i}\right)v_{j}^{e}+\rho_{j}^{i}\times \omega_{i}^{e}. \end{array}$$

To minimize the prediction error in (14), we now define the cost function as

(16) $$\begin{array}{c} J_{v_{i}}=\sum_{i=1}^{n}\frac{a_{e}}{2}∥e_{v_{i}}{∥}^{2}+\sum_{\begin{array}{c} j=1\\ \left(i\neq j\right) \end{array}}^{N}\frac{a_{r}}{2}(∥{\hat{\rho }}_{j}^{i}∥-∥{\hat{\rho }}_{i}^{j}{∥)}^{2}, \end{array}$$

where $a_{e}$ and $a_{r}$ are non-negative weights. Recalling that ${\hat{\rho }}_{j}^{i}$ is the distance to from the *i*-th end-effector to the *j*-th end-effector estimated by *i*-th aerial manipulator. Therefore, $|\rho_{j}^{i}\left|=\right|\rho_{i}^{j}|=\rho$ in the true value, which means that the relative distance between *i* and *j*-th end-effector should be the same.

In the cost function, the error term $∥e_{v_{i}}{∥}^{2}$ is designed to minimize the error and estimate ${\hat{\rho }}_{j}^{i}$. The term $(∥{\hat{\rho }}_{j}^{i}∥-∥{\hat{\rho }}_{i}^{j}{∥)}^{2}$ is the consensus term to share the same estimation results. Here, we apply the gradient rule to obtain the update rule for $\rho_{j}^{i}$ as

(17) $$\begin{array}{cc} {}^{k+1}{\hat{\rho }}_{j}^{i}= & {}^{k}{\hat{\rho }}_{j}^{i}-\alpha \frac{\partial J_{v_{i}}}{\partial {\hat{\rho }}_{j}^{i}}\\ = & {}^{k}{\hat{\rho }}_{j}^{i}-\alpha \cdot a_{e}\left(v_{i}^{e}-R\left({\hat{\psi }}_{j}^{i}\right)v_{j}^{e}-\omega_{i}^{e}\times^{k}{\hat{\rho }}_{j}^{i}\right)\\ \; & -\alpha \cdot a_{r}\sum_{\begin{array}{c} j=1\\ \left(i\neq j\right) \end{array}}^{N}{(∥}^{k}{\hat{\rho }}_{j}^{i}{∥-∥}^{k}{\hat{\rho }}_{i}^{j}∥)\frac{{}^{k}{\hat{\rho }}_{j}^{i}}{{∥}^{k}{\hat{\rho }}_{j}^{i}∥}, \end{array}$$

where α is the learning rate. Note that the kinematic estimation of ${\hat{\rho }}_{ij}$ converges to the true value ρ if the following PE (persistence of excitation) condition is satisfied as

(18) $$\begin{array}{c} \sum_{k}^{k+T}S{\left(\omega_{i}^{e}\right)}^{T}S\left(\omega_{i}^{e}\right)>0, \end{array}$$

for the constant time *T*. Here, $S(\omega_{i}^{e})$ is the skew-symmetric matrix of $\omega_{i}^{e}$. Equation (18) implies the condition of persistent excitation (PE) and the PE term should be positive definite. The estimation performance comparison will be shown in Section 5.

### 3.2. Path Planning

**Figure 3:**
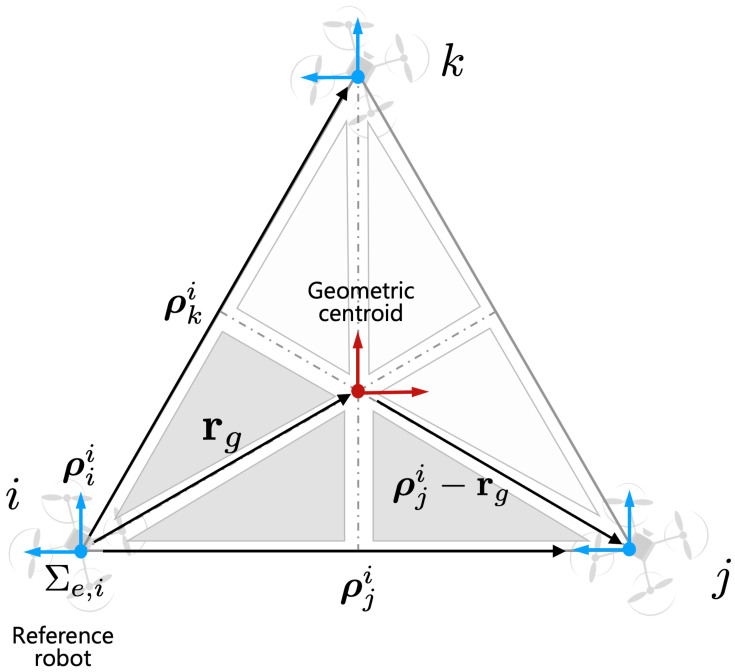
Relative vectors with respect to the reference robot in $\Sigma_{e,i}$.

As we discussed the assumption in Section 3, we assume that cooperative robots know the configuration of the payload. For example, as shown in Figure 3, aerial manipulators know the basic configuration between each robot. In this case, geometric centroid can be estimated using the estimated distance $\rho_{j}^{i}$. For simplicity of the path planning, the *i*-th robot can be considered a leader robot and the rest of the robots can be considered as follower robots. The geometric centroid $r_{g}$ can be calculated as

(19) $$\begin{array}{c} r_{g}=\sum_{\begin{array}{c} j=1\\ i\neq j \end{array}}^{N}\rho_{j}^{i}/N. \end{array}$$

Given the desired trajectory of the payload $q_{o}^{d}$, the desired trajectory for each end-effector (i.e., $q_{e,i}^{d}\in R^{6}$) is derived as

(20) $$\begin{array}{cc} q_{e,i}^{d} & =q_{o}^{d}-r_{g}\\ q_{e,j}^{d} & =q_{o}^{d}+\left(\rho_{j}^{i}-r_{g}\right)\;\left(j=1,...,N,\;i\neq j\right) \end{array}$$

where *j* is a randomly selected *j*-th aerial robot frame to unify and express it in one coordinate system among several robots (See Figure 3 for the details). Using $q_{e,i}^{d}$, the desired trajectory for each aerial manipulator $q_{e,i}^{d}\in R^{8}$ is obtained based on the inverse kinematics solution. The details of inverse kinematics are described in [4].

## 4. Dynamic Parameter Estimation

External wrench estimation to handle unexpected collisions of robots has begun with early works on stationary ground manipulators [25]. To increase the speed and accuracy of the calculation, the wrench estimation algorithm is newly developed for a dual-arm manipulator [26] and now it is also applied to an aerial robot [15,16]. The hybrid estimation algorithm based on a generalized momentum and acceleration was satisfactorily applied for an aerial robot to detect collisions [15,16]. However, these algorithms [15,16] are applied only for a single aerial robot and the estimation performance was not verified in the presence of sensor noise. Research has been studied to solve the sensor noise problem of the wrench estimation algorithm [27], but it was applied only to the collision problem of a single ground manipulator.

To handle the aforementioned issue, in this section, we propose the dynamic parameter estimator for cooperative aerial manipulators to obtain the payload mass ${\hat{m}}_{o}$ and $r_{i}$. If a payload has uniform mass distribution and the aerial manipulators exert the same force on the grasping point of the payload, then the $r_{i}$ is easy to compute, and the mass ${\hat{m}}_{o}$ can be estimated only using only the state variable of a robot itself as described in our previous work [4]. However, for the payload with non-uniform mass distribution, previous works cannot be applied because the geometry centroid and COM of the payload are different. To overcome this issue, in this paper, we design the dynamic-parameter-updates rule for the cooperative aerial manipulator by exploiting the external wrench estimation algorithm.

In this paper, as configuration of a payload is given as described in Section 3.1, we can assume that the moment of inertia is approximated using the kinematic (i.e., $\rho_{j}^{i}$) and dynamic parameter (i.e., $m_{o}$ and $r_{i}$) of a payload [28]. Therefore, we focus on the estimation of $m_{o}$ and $r_{i}$.

### 4.1. First-Order Momentum Observer

Before addressing the proposed parameter estimation algorithm, we briefly explain the classical first-order momentum-based wrench estimator for the performance comparison used in simulations as used in [14].

The momentum-based estimator is designed based on the generalized momentum of the robot as

(21) $$\begin{array}{cc} p_{i} & =D_{i}{\dot{q}}_{i}. \end{array}$$

Using (21), the time derivative of $p_{i}$ is given as

(22) $$\begin{array}{cc} {\dot{p}}_{i} & =\tau_{i}+\tau_{i}^{b}-C_{i}{\dot{q}}_{i}-G_{i}+\dot{D}{\dot{q}}_{i}\\ \; & =\tau_{i}+\tau_{i}^{b}+C_{i}^{T}{\dot{q}}_{i}-G_{i}. \end{array}$$

Here, the passivity property (i.e., $\dot{D}\left(q_{i}\right)=C\left(q_{i},{\dot{q}}_{i}\right)+C^{T}\left(q_{i},{\dot{q}}_{i}\right)$) was used. $\tau_{i}^{b}$ and is the external wrench that we want to estimate.

The derivation of classic momentum-based estimator is designed based on the following residual vector as

(23) $$\begin{array}{c} m_{i}=K_{o}\left(p_{i}-\int \left(\tau_{i}+C_{i}^{T}{\dot{q}}_{i}-G_{i}+m_{i}\right)ds-p_{i}\left(0\right)\right), \end{array}$$

where p(0) is the initial value of *p* and $K_{o}$ is the positive gain. By using (22), the time derivative of the residual **r** can be obtained as

(24) $$\begin{array}{c} {\dot{m}}_{i}=-K_{o}\left(m_{i}-\tau_{i}^{b}\right). \end{array}$$

This equation means that $m_{i}$ is the first-order filtered value of $\tau_{i}^{b}$; therefore, it can be used as an estimation of the external wrench.

### 4.2. Dynamic Parameter Estimation with Sliding Mode Momentum Observer

In the classical momentum-based observer, to achieve $m_{i}\approx \tau_{i}^{b}$, $K_{o}$ should be large enough. However, a large value of $K_{o}$ results in higher noise amplification in the estimated wrench. In this case, the estimation performance of dynamic parameters may deteriorate. This issue can be solved by exploiting the second-order sliding mode (SOSM) momentum observer which was introduced in [29].

SOSM observer, the so-called super twisting algorithm, is widely used in sliding-mode literature to design controllers, observers, and exact differentiators. Using the generalized momentum in (21), the momentum observer can be designed

(25) $$\begin{array}{cc} {\dot{\hat{p}}}_{i} & =\tau_{i}+C_{i}^{T}{\dot{q}}_{i}-G_{i}-T_{1}\left|{\tilde{p}}_{i}\right|-T_{2}{\tilde{p}}_{i}+\sigma_{i} \end{array}$$

(26) $$\begin{array}{cc} {\dot{\sigma }}_{i} & =-S_{1}\mathrm{sgn}\left({\tilde{p}}_{i}\right)-S_{2}{\tilde{p}}_{i}, \end{array}$$

where ${\hat{p}}_{i}$ is the estimated momentum of *i*-th aerial manipulator and ${\tilde{p}}_{i}={\hat{p}}_{i}-p_{i}$. $S_{i}$ and $T_{i}$ are the user-defined gain.

Using (22) and the definition of new variable $s_{i}=\sigma_{i}-\tau_{i}^{b}$, we can obtain the observer error dynamics as

(27) $$\begin{array}{cc} {\dot{\tilde{p}}}_{i} & =-T_{1}{\left|{\tilde{p}}_{i}\right|}^{1/2}\mathrm{sgn}\left({\tilde{p}}_{i}\right)-T_{2}{\tilde{p}}_{i}+\sigma_{i}\\ {\dot{s}}_{i} & =-S_{1}\mathrm{sgn}\left({\tilde{p}}_{i}\right)-S_{2}{\tilde{p}}_{i}-{\dot{\tau }}_{i}^{b}. \end{array}$$

**Figure 4:**
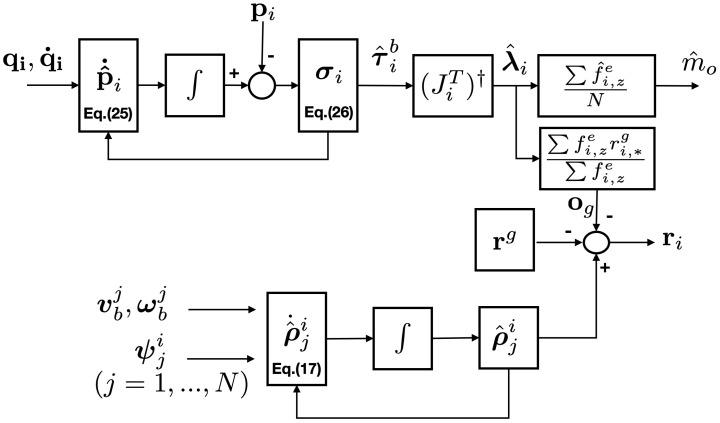
The proposed structure for the dynamic parameter estimation.

Assuming that $\tau_{i}^{b}$ and $\sigma_{i}$ are globally bounded by some known constant and $S_{i}$ and $T_{i}$ are selected high enough, then the origin $\left({\tilde{p}}_{i},s_{i}\right)=\left(0,0\right)$ is an equilibrium point that is globally asymptotically stable. Here, we can say that $\sigma_{i}$ becomes an estimation of $\tau_{i}^{b}$. The detailed proofs are described in [29]. The detailed process for dynamic parameter estimation is described in Figure 4.

As $\tau_{i}^{b}\in R^{8}$ is the external wrench applied to the aerial manipulator in $\Sigma_{b,i}$, we have to compute the applied force at the end-effector $\lambda_{i}$. Following the description in [30], $\lambda_{i}$ can be derived as

(28) $$\begin{array}{cc} {\hat{\lambda }}_{i} & ={\left(J_{i}^{T}\right)}^{†}{\hat{\tau }}_{i}^{b}\\ {}[{\hat{f}}_{i,x}^{e},{\hat{f}}_{i,y}^{e},{\hat{f}}_{i,z}^{e}{]}^{T}: & =\left[\begin{array}{cc} I_{3} & 0_{3} \end{array}\right]{\hat{\lambda }}_{i} \end{array}$$

where $I_{3}$ and $0_{3}$ are 3×3 identity and zero matrix, respectively. ${\left(J_{i}^{T}\right)}^{†}={\left(J_{i}J_{i}^{T}\right)}^{-1}J_{i}$ is the pseudo inverse of $J_{i}^{T}$. Here, we only need the force at x,y and *z* direction, so $\left[\begin{array}{cc} I_{3} & 0_{3} \end{array}\right]$ is added.

Now, the dynamic parameters (i.e., ${\hat{m}}_{o}$ and $r_{i}$) in Figure 1 are estimated using the estimated wrench in (). The mass is updated as

(29) $$\begin{array}{c} {\hat{m}}_{o}=\frac{\sum_{i=1}^{N}{\hat{f}}_{i,z}^{e}}{N\times g}, \end{array}$$

where *g* is gravitational constant and ${\hat{f}}_{i,z}^{e}$ is the estimated force by *i*-th aerial manipulator at *z*-direction.

The momentum generated by the effective force at the end-effector should be zero, so the following equation should be satisfied:

(30) $$\begin{array}{c} \sum_{j=1}^{N}\left(\rho_{j}^{i}-r_{g}-o_{g}\right)\times {\hat{f}}_{j}^{e}=0. \end{array}$$

**Figure 5:**
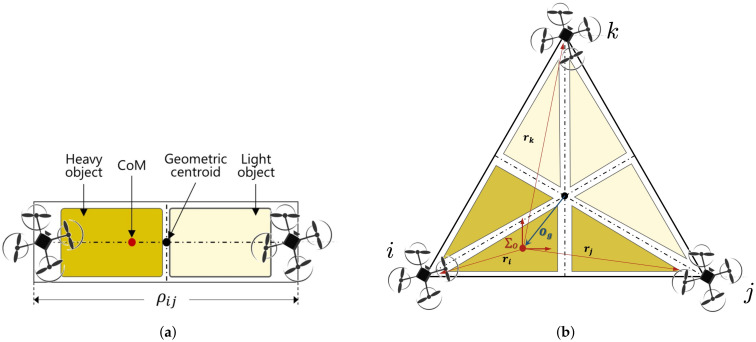
Analysis for the center of mass of the common payload: (**a**) by two aerial manipulators and (**b**) by three aerial manipulators.

Note that *i* in (30) refers to the leader robot or the reference frame. As this paper considers the flat-surface payload as shown in Figure 5, Equation (30) can be rewritten as

(31) $$\begin{array}{cc} \; & \sum_{j=1}^{N}{\hat{f}}_{j,z}^{e}\left(r_{j,y}^{g}-o_{g,y}\right)=0,\sum_{j=1}^{N}{\hat{f}}_{i,z}^{e}\left(r_{j,x}^{g}-o_{g,x}\right)=0 \end{array}$$

(32) $$\begin{array}{cc} \; & \sum_{j=1}^{N}{\hat{f}}_{j,y}^{e}\left(r_{j,x}^{g}-o_{g,x}\right)-{\hat{f}}_{j,x}^{e}\left(r_{j,y}^{g}-o_{g,y}\right)=0 \end{array}$$

Finally, $o_{x}$ and $o_{y}$ are computed as

(33) $$\begin{array}{c} o_{x}=\frac{\sum {\hat{f}}_{j,z}^{e}r_{j,x}^{g}}{\sum {\hat{f}}_{j,z}^{e}},o_{y}=\frac{\sum {\hat{f}}_{j,z}^{e}r_{j,y}^{g}}{\sum {\hat{f}}_{i,z}^{e}}. \end{array}$$

Now, we can compute $r_{i}$ as $r_{i}=\left(\rho_{j}^{i}-r_{g}-o_{g}\right)$. Equation (32) may not be necessary for the flat-surface payload considered in this paper, but it is necessary for verification of the estimated value. In addition, if the surface of the payload is not flat, $o_{g}$ can be estimated by solving (30) directly.

## 5. Simulations

To evaluate the performance of the proposed algorithm, we present several simulation results to compare the performances of the proposed algorithm with the conventional algorithm. As the conventional estimation algorithm for cooperative robots [4,9,13,14] is difficult to handle the payload with an arbitrary COM well, it is hard to compare the estimation performance with the proposed algorithm. For this reason, we divide performance comparison into two fields, mass estimation, and kinematic parameter estimation, and compare them with existing algorithms. The mass estimation performance is compared with first-order-based momentum observer [14] and our previous parameter estimation algorithm [4]. For the kinematic parameter estimation, the adaptive control for an inaccurate kinematic model [10] is used. For detailed analysis, two scenarios (two drones or three drones) as shown in Figure 5 are considered in the simulation.

### 5.1. Simulation Environment

In the simulations, each aerial manipulator consists of a quadrotor and a 2-DOF arm. For the two aerial manipulators in Figure 5a, the mission consists of manipulating an unknown payload from the initial position ${\left[0,0.6,0.6\right]}^{T}$ m to ${\left[1.2,1.6,0.6\right]}^{T}$ m. The payload is characterized as $m_{o}=0.5$, $o_{x}=0$ m, and $o_{y}=-0.2$ m. The total length of the payload is l:=0.8 m. For the three aerial manipulators in Figure 5b, the initial position of the payload is ${\left[0.23,0.4,0.6\right]}^{T}$ m and the final position of the payload is ${\left[1.73,1.9,0.6\right]}^{T}$ m. In this mission, the payload is characterized as $m_{o}=0.5$, $o_{x}=0.1$ m, and $o_{y}=0.1$ m. In the triangular payload, the length of each side is set to 0.8 m.

In both scenarios, for the property of the persistence of excitation (PE) in (18), two aerial manipulators rotate for 5 s. After the PE process is finished, the cooperative aerial manipulator follows the desired trajectory.

The control input in (8) is calculated as

(34) $$\begin{array}{c} \tau_{i}={\hat{D}}_{i}{\ddot{q}}_{r,i}+{\hat{C}}_{i}{\dot{q}}_{r,i}+{\hat{G}}_{i}-\left(\kappa_{s,i}+\delta_{i}\right)s_{i} \end{array}$$

where ${\hat{D}}_{i}$, ${\hat{C}}_{i}$, and ${\hat{G}}_{i}$ include the estimated payload mass ${\hat{m}}_{o}$. $\kappa_{s,i}$ is the positive-definite gain matrix. Here, $\delta_{\tau }$ is the auxiliary control input for handling properties and defined as

(35) $$\begin{array}{c} \delta_{i}:=\frac{c_{i}}{2}J_{e,i}^{T}E_{i}^{†}\left({\dot{H}}_{o}-2\mu \right)E_{i}^{-T}J_{e,i}. \end{array}$$

The sliding surface variable $s_{i}$ can be defined as

(36) $$\begin{array}{cc} {\dot{q}}_{r,i} & ={\dot{q}}_{i}^{d}-\Lambda_{i}e_{i}\\ s_{i} & ={\dot{q}}_{i}-{\dot{q}}_{r,i}={\dot{e}}_{i}+\Lambda_{i}e_{i} \end{array}$$

where $\Lambda_{i}$ is a positive diagonal matrix and $q_{i}^{d}$ is the desired state of the *i*-th aerial manipulator. The detailed process for the controller is described in our previous research [4].

For the performance analysis, we assumed that

(1) The main uncertainty is caused by the measurement noise, ${\dot{q}}_{i}^{mes}={\dot{q}}_{i}+\epsilon_{v},q_{i}^{mes}=q_{i}+\epsilon_{p}$.
(2) The noise in the measured state $\epsilon_{v}$ and $\epsilon_{p}$ has a high frequency and is zero mean.

In the simulations, the standard deviation of the measurement noise $\epsilon_{v}$ and $\epsilon_{p}$ are set as

- 0.2 m in *x* and *y* direction and 0.1 m in the *z* direction
- 0.01 rad in attitude and 0.1 rad in joint angles.

Let ${\hat{\epsilon }}_{k}$ is the estimated parameter of ϵ at the time step *k* and ϵ is the true value. The performance of the payload mass (i.e., ${\hat{m}}_{o}$) and kinematic parameter estimation (i.e., ${\hat{\rho }}_{ij}$) is evaluated by considering the average error ($E_{ae}$) and the maximum error $E_{me}$:

(37) $$\begin{array}{c} E_{ae}=\frac{1}{N}\sum_{k=1}^{T}∥{\hat{\epsilon }}_{k}-\epsilon ∥,E_{me}=\max_{k}∥{\hat{\epsilon }}_{k}-\epsilon ∥. \end{array}$$

To analyze the above performance, Monte Carlo simulations are also performed using 30 sample runs.

**Figure 6:**
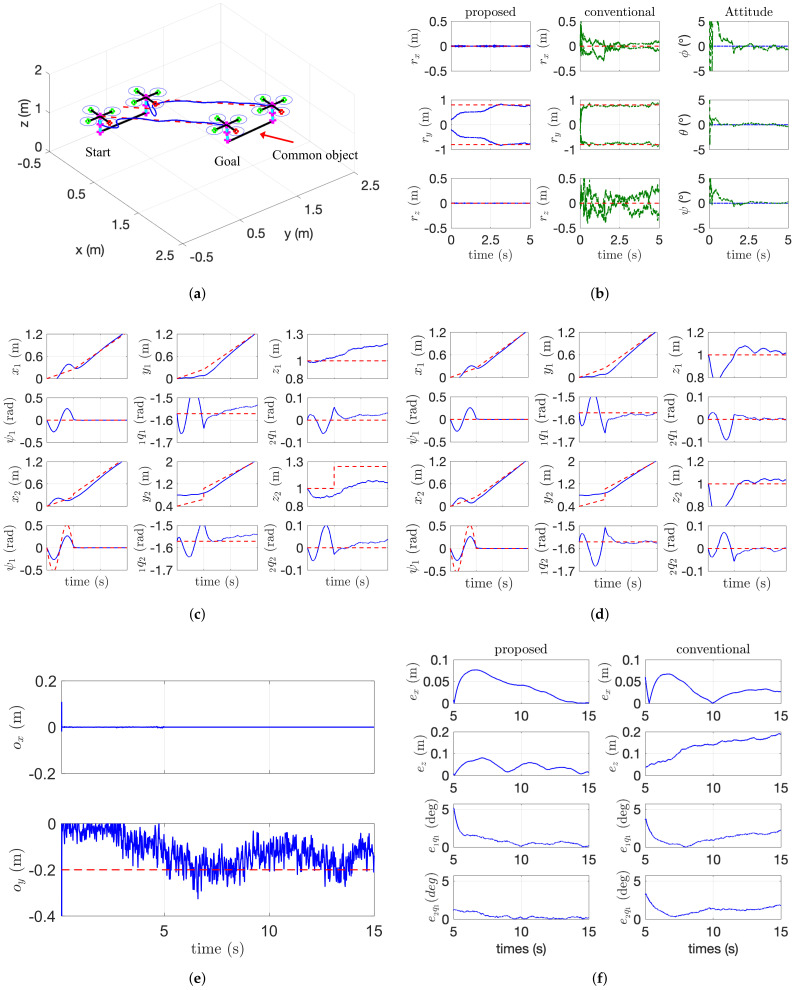
Trajectory tracking of a cooperative aerial manipulators. (**a**) 3D flight scenario. (**b**) Kinematic parameter estimation. (**c**) by the conventional algorithm. (**d**) by the proposed algorithm. (**e**) Estimation of $o_{x}$ and $o_{y}$. (**f**) Tracing error comparison.

Based on our proposed framework discussed in the previous section, we first conducted a simulation study with two aerial manipulators carrying an irregular payload, which are shown in Figure 6a. During the phase to satisfy PE condition (18), the desired state for the first aerial manipulator is fixed and the desired state for the second aerial manipulator is set as

(38) $$\begin{array}{c} q_{2}^{d}={\left[0.05\times t,0.05\times t,0.6,0,0,-30\sin\left(2\pi /5t\right),-90,0\right]}^{T}, \end{array}$$

where unit of the attitude and joint angles is written in degrees. After the kinematic estimation phase, the desired trajectory for the payload is set as

(39) $$\begin{array}{c} q_{o}^{d}={\left[0.1\times t,0.1\times t,0.6,0,0,0\right]}^{T}. \end{array}$$

We assume that the noises for the translational and angular velocities have a value that is twice as large as the position noise.

### 5.2. Simulation Results

**Figure 7:**
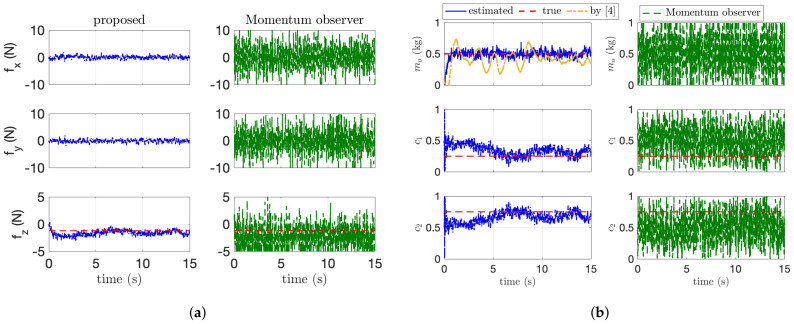
Dynamic parameter estimation. (**a**) Force estimation results. (**b**) Dynamic parameter estimation.

The simulation data are shown in Figure 6 and Figure 7. In Figure 6, the comparison results including kinematic parameter estimation and trajectory tracking performance are shown. In Figure 6b, the red dotted line is the true value and the blue line is the estimated results by the proposed algorithm, and the green dotted line is the estimated results by [10]. The estimation error between the proposed method and conventional method in Figure 6b is also caused by the fact that the algorithm proposed in [10] estimates six-DOF poses of a payload while the proposed algorithm in this paper estimates only four-DOF poses of the payload considering the characteristics of the cooperative manipulator. For this reason, we can accurately estimate the location of COM (i.e., $o_{x}$ and $o_{y}$) in the noisy measurement as shown in Figure 6e. In Figure 6c,d, the red-dotted line is the desired state for the aerial manipulator and the blue line means the actual states. As the conventional estimation algorithm for the relative distance $\rho_{j}^{i}$ contains a relatively large error, the desired trajectory in the *x* and *z* direction may incorrectly be adjusted. The tracking performance in *x* and *z* direction also affects the joint angle of the arm. So our proposed algorithm shows better tracking performance as shown in Figure 6f.

The dynamic parameter estimation is addressed in Figure 7. In Figure 7, the blue and green line is the estimated value by the proposed algorithm and the first-order-based momentum observer used in [14]. The orange line in Figure 7b is the estimated mass by our previous research in [4]. Our previous algorithm [4] for the mass estimation shows better performance compared with the first-order momentum observer [14], but the proposed method in this paper shows the best performance. This is because our algorithm with second-order sliding-mode observer accurately estimates the external wrench applied to the end-effector than the first-order-based momentum observer as shown in Figure 7a. Although showing better performance than the momentum-based observer, our previous work cannot handle the payload with an arbitrary COM because of the assumption of the uniform-mass distribution. In addition, as the force distribution $c_{i}$ in (5) can be calculated using the estimated force on *z* axis (i.e., $c_{i}={\hat{f}}_{i,z}^{e}/\sum_{j=1}^{N}{\hat{f}}_{j,z}^{e}$), the estimation of $c_{i}$ in Figure 7b describes that our proposed algorithm estimates the applied wrench precisely. The detailed estimation performance for the dynamic and kinematic parameters is presented in Table 2. In this table, the average error $E_{ae}$ and the maximum error $E_{me}$ are calculated after parameter converges. std in Table 2 means standard deviation. As described in Table 2, the proposed algorithm has the smallest value in both $E_{ae}$ and $E_{me}$.

**Figure 8:**
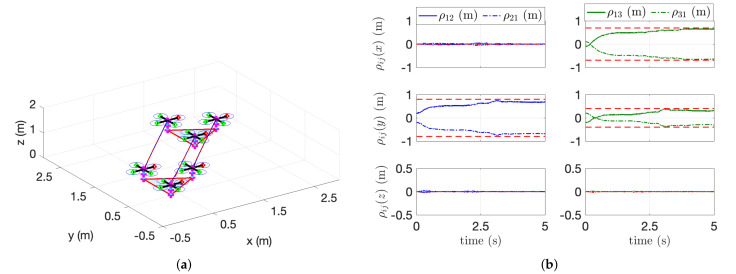
Trajectory tracking of three cooperative aerial manipulators. (**a**) 3D fight of three aerial manipulators. (**b**) Estimation of relative distances ($\rho_{ij}$).

**Figure 9:**
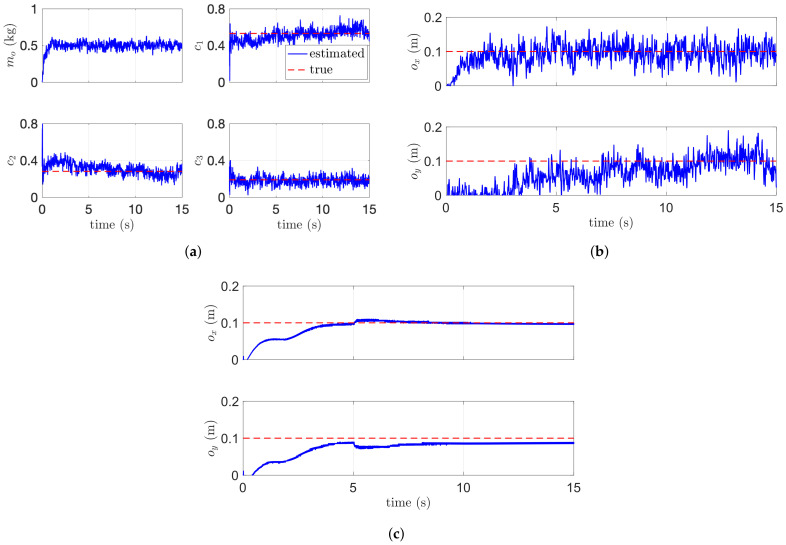
Estimation performance of three aerial manipulators. (**a**) Dynamic parameter estimation. (**b**) Estimation of $o_{x}$ and $o_{y}$. (**c**) Estimation of $o_{x}$ and $o_{y}$ without noise.

The second simulation using three aerial manipulators is described in Figure 8 and Figure 9. Unlike the first scenario with two aerial manipulators, the leader or reference aerial manipulator in the second scenario estimates $\rho_{j}^{i}$ (e.g., i=1, and j=2,3) simultaneously using their velocity measurement. In this case, it is difficult to distinguish whether the effect in (14) is caused by the robot with j=2 or the robot with j=3. To overcome this issue, the consensus parameter $a_{r}$ in (17) for the reference or leader robot is set to 0. For the follower robot, we set $a_{e}\neq 0$. Figure 8b shows the estimation performance. The relative distances between *i*-th and *j*-th aerial manipulators are estimated precisely even in the complex-shaped payload. Figure 9 shows the estimation of the mass and the COM of the payload. Our algorithms estimate the COM of the payload satisfactorily with or without the noisy measurement.

## 6. Conclusions

This paper presents a cooperative aerial manipulation algorithm to handle a payload with an arbitrary center-of-mass. The proposed framework consists of two estimation algorithms: relative distance estimation and dynamic parameter estimation. The relative distances are estimated using the translational and angular velocities between aerial robots. By exploiting the distance estimation, the desired trajectories for each aerial manipulator are set. After the kinematic estimation, the dynamic parameter estimation is performed for the mass of the common object and the vector between the end-effector frame and the COM of the payload. We performed flight simulations using multiple aerial manipulators and compared the proposed algorithm with the conventional method involving our previous work or the first-order momentum-based algorithm. The simulation results showed that the proposed algorithm achieved the best performance in parameter estimation and trajectory tracking.

Our future works include relaxation of PE (persistence of excitation) conditions and taking into account complex payload types. The relaxation of PE is required for the safety of drones because it can be dangerous when the drone rotates to satisfy the PE condition. In addition, the proposed method assumes that the COM and the geometric centroid are on the same horizontal plane and the configuration of a payload should be known previously, otherwise it may be difficult to estimate. Experimental validation of the proposed method on a real setup is needed to address the above-mentioned challenges.

## Figures and Tables

**Table 1:** The major terms used in this paper.

Term	Definition
$q_{i}\in R^{8}$	a state vector for *i*-th aerial manipulator
$\tau_{i}\in R^{8}$	a control input
$\lambda_{i}\in R^{8}$	an applied force at the *i*-th end-effector
$\tau_{i}^{b}\in R^{8}$	an external wrench applied to $\Sigma_{b,i}$
$c_{i}\in R$	a force balance term
$v_{i}^{e}\in R^{3}$	a linear velocity at $\Sigma_{e,i}$
$\omega_{i}^{e}\in R^{3}$	an angular velocity at $\Sigma_{e,i}$
$m_{o}\in R$	a mass of a payload
$\psi_{j}^{i}\in R$	a relative heading angle between $\Sigma_{b,i}$ and $\Sigma_{b,j}$
$r_{i}\in R^{3}$	a vector from $\Sigma_{o}$ to $\Sigma_{e,i}$
$E_{i}\in R^{6\times 6}$	a grasp matrix
$r_{g}\in R^{3}$	the geometric centroid of the payload wr.t. the leader robot
$\rho_{j}^{i}\in R^{3}$	a relative distance from $\Sigma_{e,i}$ to $\Sigma_{e,j}$
$p_{i}\in R^{8}$	a generalized momentum
$\sigma_{i}\in R^{8}$	an estimated external wrench
$o_{x,y}\in R$	a vector from the geometric centroid to $\Sigma_{o}$

**Table 2:** Mass and kinematic parameter estimation performance.

	Proposed Algorithm		Comparison Algorithm		
**Parameter**	** $E_{\mathrm{ae}}$ ** ***(std)***	$E_{\mathrm{me}}$	** $E_{\mathrm{ae}}$ ** ***(std)***	$E_{\mathrm{me}}$	**Ref**
${\hat{m}}_{o}$ [kg]	0.1968 (0.0094)	0.1883	0.4859 (0.0134)	1.0196	by [14]
			0.3275 (0.0104)	0.4604	by [4]
${\hat{\rho }}_{j}^{i}\left(x\right)$ [m]	0.1265 (0.0107)	0.0242	0.2015 (0.0479)	0.1120	
${\hat{\rho }}_{j}^{i}\left(y\right)$ [m]	0.2456 (0.0463)	0.0363	0.2550 (0.0680)	0.1004	by [10]
${\hat{\rho }}_{j}^{i}\left(z\right)$ [m]	0.0549 (0.0055)	0.0048	0.4081 (0.1073)	0.3757	

## Data Availability

Not applicable.

## References

[1] Huang H., Savkin A.V., Huang C. (2020). Scheduling of a parcel delivery system consisting of an aerial drone interacting with public transportation vehicles. Sensors.

[2] Mellinger D., Lindsey Q., Shomin M., Kumar V. Design, modeling, estimation and control for aerial grasping and manipulation. Proceedings of the 2011 IEEE/RSJ International Conference on Intelligent Robots and Systems.

[3] Eoh G., Park T.H. (2021). Cooperative Object Transportation Using Curriculum-Based Deep Reinforcement Learning. Sensors.

[4] Lee H., Kim H., Kim W., Kim H.J. (2018). An Integrated Framework for Cooperative Aerial Manipulators in Unknown Environments. IEEE Robot. Autom. Lett..

[5] Lee H., Son C.Y., Kim H.J. (2019). Collision-Free Path Planning for Cooperative Aerial Manipulators Under Velocity and Curvature Constraints. IEEE Access.

[6] Kim S., Seo H., Shin J., Kim H.J. (2018). Cooperative Aerial Manipulation Using Multirotors With Multi-DOF Robotic Arms. IEEE/ASME Trans. Mechatron..

[7] Tagliabue A., Kamel M., Siegwart R., Nieto J. (2019). Robust collaborative object transportation using multiple mavs. Int. J. Robot. Res..

[8] Thapa S., Bai H., Acosta J.Á. (2020). Cooperative Aerial Manipulation with Decentralized Adaptive Force-Consensus Control. J. Intell. Robot. Syst..

[9] Marino A., Pierri F. (2018). A two stage approach for distributed cooperative manipulation of an unknown object without explicit communication and unknown number of robots. Robot. Auton. Syst..

[10] Aghili F. (2013). Adaptive Control of Manipulators Forming Closed Kinematic Chain With Inaccurate Kinematic Model. IEEE/ASME Trans. Mechatron..

[11] Pierri F., Nigro M., Muscio G., Caccavale F. (2020). Cooperative Manipulation of an Unknown Object via Omnidirectional Unmanned Aerial Vehicles. J. Intell. Robot. Syst..

[12] Suarez A., Heredia G., Ollero A. (2018). Physical-Virtual Impedance Control in Ultralightweight and Compliant Dual-Arm Aerial Manipulators. IEEE Robot. Autom. Lett..

[13] Franchi A., Petitti A., Rizzo A. Decentralized parameter estimation and observation for cooperative mobile manipulation of an unknown load using noisy measurements. Proceedings of the 2015 IEEE International Conference on Robotics and Automation (ICRA).

[14] Ruggiero F., Cacace J., Sadeghian H., Lippiello V. (2015). Passivity-based control of VToL UAVs with a momentum-based estimator of external wrench and unmodeled dynamics. Robot. Auton. Syst..

[15] Tomić T., Ott C., Haddadin S. (2017). External Wrench Estimation, Collision Detection, and Reflex Reaction for Flying Robots. IEEE Trans. Robot..

[16] Ryll M., Muscio G., Pierri F., Cataldi E., Antonelli G., Caccavale F., Bicego D., Franchi A. (2019). 6D interaction control with aerial robots: The flying end-effector paradigm. Int. J. Robot. Res..

[17] Yang H., Lee D. Hierarchical cooperative control framework of multiple quadrotor-manipulator systems. Proceedings of the 2015 IEEE International Conference on Robotics and Automation (ICRA).

[18] Korayem A.H., Nekoo S.R., Korayem M.H. (2019). Optimal sliding mode control design based on the state-dependent Riccati equation for cooperative manipulators to increase dynamic load carrying capacity. Robotica.

[19] Ćehajić D., gen Dohmann P.B., Hirche S. Estimating unknown object dynamics in human-robot manipulation tasks. Proceedings of the 2017 IEEE International Conference on Robotics and Automation (ICRA).

[20] Morín D.G., Araujo J., Tayamon S., Andersson L.A.A. Autonomous Cooperative Flight of Rigidly Attached Quadcopters. Proceedings of the 2019 International Conference on Robotics and Automation (ICRA).

[21] Erhart S., Hirche S. (2016). Model and Analysis of the Interaction Dynamics in Cooperative Manipulation Tasks. IEEE Trans. Robot..

[22] Lee H., Kim H., Kim H.J. (2018). Planning and Control for Collision-Free Cooperative Aerial Transportation. IEEE Trans. Autom. Sci. Eng..

[23] Montijano E., Cristofalo E., Zhou D., Schwager M., Sagüés C. (2016). Vision-Based Distributed Formation Control Without an External Positioning System. IEEE Trans. Robot..

[24] Cantieri A., Ferraz M., Szekir G., Antônio Teixeira M., Lima J., Schneider Oliveira A., Aurélio Wehrmeister M. (2020). Cooperative UAV–UGV Autonomous Power Pylon Inspection: An Investigation of Cooperative Outdoor Vehicle Positioning Architecture. Sensors.

[25] De Luca A., Mattone R. Sensorless Robot Collision Detection and Hybrid Force/Motion Control. Proceedings of the 2005 IEEE International Conference on Robotics and Automation.

[26] Han L., Xu W., Li B., Kang P. (2019). Collision Detection and Coordinated Compliance Control for a Dual-Arm Robot Without Force/Torque Sensing Based on Momentum Observer. IEEE/ASME Trans. Mechatron..

[27] Garofalo G., Mansfeld N., Jankowski J., Ott C. Sliding Mode Momentum Observers for Estimation of External Torques and Joint Acceleration. Proceedings of the 2019 International Conference on Robotics and Automation (ICRA).

[28] Hibbeler R.C. (2004). Engineering Mechanics: Statics.

[29] Moreno J.A., Osorio M. A Lyapunov approach to second-order sliding mode controllers and observers. Proceedings of the 2008 47th IEEE Conference on Decision and Control.

[30] Wahrburg A., Robertsson A., Matthias B., Dai F., Ding H. Improving contact force estimation accuracy by optimal redundancy resolution. Proceedings of the 2016 IEEE/RSJ International Conference on Intelligent Robots and Systems (IROS).

